# News and Coming Events

**Published:** 1907-10-05

**Authors:** 


### NEWS AND COMING EVENTS.

The Lord Mayor will preside at the 68th Annual Festival of the Newsvendors' Benevolent and Provident Institution on Tuesday, October 22, at De Keyser's Royal Hotel.

The annual students' dinner of University College Hospital was held in the Library of the Medical School Buildings, entrance in University Street, on Wednesday, October 2, at 7 for 7.30, Sir William R. Gowers, F.R.C.P., F.R.S., in the chair.

Sir Frederic Treves, Bart., G.C.V.O., C.B., LL.D., will distribute the prizes to successful students of the Royal Dental Hospital, London, on Friday, October 13, at 8 p.m. precisely, at the Royal Institute Galleries, Princes Hall, Piccadilly.

The Government of the United States has officially invited Professors Metchnikoff, Calmette, and Letulle, of Paris, to take part in the deliberations of the International Congress on Tuberculosis which is to be held in Washington in 1908.

Colonel F. J. Lambkin, Lecturer in Syphilology at the Royal Army Medical School, is proceeding on a special mission to the Uganda Protectorate, at the instance of the Secretary of State, to report on the prevalence of syphilis in the Protectorate and to suggest measures for checking its ravages.

The London Medical Exhibition organised by *The British and Colonial Druggist*, will be opened on Monday, October 7 next. The exhibits are arranged in the Royal Horticultural Hall, Vincent Square, Westminster, and will be on view from 11 a.m. till 7 p.m. from the opening day until October 11 next.

The new operating-theatre of the Longton Accident Hospital was opened early in September. The theatre has been built at a cost of nearly £1,250, and is a thoroughly efficient working room. Many of the fittings were presented by friends of the institution. Thus the excellent operating-table is the gift of Mr. Thomas Forester, while the instrument-case was presented by Mr. John Brunt. An efficient sterilising-plant has been fitted up in the theatre, and a small but excellently arranged *x*-ray installation has been added.

The session of University College Hospital Medical School was opened by a formal ceremony on Wednesday, October 2, at three o'clock, when an introductory address was delivered by Sir Richard Douglas Powell, Bart., K.C.V.O., D.Sc., M.D., P.R.C.P., Physician in Ordinary to his Majesty the King. At the conclusion of the address medallion portraits of the late Professor Christopher Heath, LL.D., F.R.C.S., and of the late Professor G. V. Poore, M.D., F.R.C.P., were unveiled in the entrance-hall of the school.

The Preston, Fulwood, and Longridge Joint Hospital was formally opened on September 4 by Dr. C. J. Trimble, J.P., C.M.G., of Brownedge, in the presence of a large gathering of ladies and gentlemen. The buildings so far completed fully are the administration block, the scarlet fever block, and the isolation pavilion, but four additional scarlet fever blocks, together with a typhoid and diphtheria block, are in course of construction. The four wards opened contain twenty-six beds, twenty for scarlet fever cases, four for enteric fever, and two for special cases. The total cost of the scheme has been bout £16,000.

The inaugural address of the winter session of the London School of Tropical Medicine, Connaught Road, Albert Dock, E., will be delivered in the lecture theatre of the School by Sir Lauder Brunton, M.D., LL.D., on Monday, October 21, 1907. The chair will be taken at three o'clock precisely by R. L. Antrobus, Esq., C.B., Assistant Under Secretary of State for the Colonies. The school and hospital will be open for inspection.

Probably the last piece of work done by the late Sir William Broadbent, Bart., was in connection with his editorship of the "Standard Family Physician," which Messrs. Funk and Wagnalls Company have now in the press. This cyclopædia will be published in November, and consists of three large volumes with more than 500 illustrations and diagrams, including an anatomical mannikin.

Miss Helen Keller, who is blind, deaf, and dumb, contributes an interesting article to the August issue of "The World's Work," her theme being the multiplicity of systems of typography for the use of the blind. Miss Keller condemns such multiplicity as being wasteful, extravagant, and totally unnecessary. "For my part," she writes, "I wish nothing had been invented except European Braille."

The Annual British Homœopathic Congress was held at Harrogate on September 19. The meetings were held at the Hôtel Majestic, and there was a fairly representative gathering of the followers of Hahnemann. The President, Dr. W. T. P. Wolston, of Edinburgh, delivered an interesting address dealing with the subject of "Spas I Have Seen." His remarks were of an eminently practical character. As, however, he very truly said, "At first sight it might not seem that hydro-therapeutic treatment had much, if anything to do with homœopathy." He qualified this remark by expressing his opinion that the curative effects of these waters lie not so much in the massive doses of the ordinary salts which they contain, and which are usually prescribed, but in the almost infinitesimal quantities of such deeply-seated drugs like bromide, iodine, etc. Dr. Wolston stated that for the past thirty years he had spent his annual holidays in visiting the various health resorts and spas, both of Great Britain and the Continent, with the special object of making himself acquainted with the climate, hygienic, and, where existing, the balneological conditions which characterised these health resorts. He urged his colleagues to do the same and expressed his opinion that too often patients were recommended by their medical advisers to go to continental spas respecting which they—the doctors—were entirely ignorant, except from what they had read in articles written by persons who had an interest in some particular spa. Dr. Wolston referred to the principal British spas, taking Harrogate first, which place, he said, rightly claimed to hold a foremost place in our list of health resorts, both on account of its numerous springs, its complete installation of baths of every description, its absence of humidity, and its bracing air. After referring in detail to the special characteristics of such places as Bath, Buxton, Strathpeffer, and other British spas he dealt with the principal continental resorts. Other papers were read by Dr. Wilde and Dr. Madden, and in the afternoon the members inspected the Royal baths and pump rooms, under the guidance of Mr. H. J. Buckland, the general manager of all the municipal bath and water establishments, after which they were driven out over the moors to visit the Fewston reservoirs In the evening the members dined together at the Hôtel Majestic, when a piece of plate was presented to Dr. Dyce-Brown in recognition of his twenty-five years' services to British homœopathy.

